# 

**Published:** 1887-05

**Authors:** 


					﻿ISS’7.
OLD SERIES
VoU XXV
I8S5 'd'
NEW SERIES
Vol. IV.-No 3. ♦


jniffiNAB


^SHwrO
1
W.F.WESfMOR£E^H
U.VM.MILLER.M.DJ.L .D.JE
JAMES A.GRAY, MXI.1
W	-----5>Ji
-/uBLlSHED By JAMES PHARRISQN&COfl
«2Ltlanta.ga.
SUBSCRIPTIONS S2.BO A. YEAR.
				

